# Impact on emergency and elective hospital-based care in Scotland over the first 12 months of the pandemic: interrupted time-series analysis of national lockdowns

**DOI:** 10.1177/01410768221095239

**Published:** 2022-05-03

**Authors:** Syed Ahmar Shah, Rachel H Mulholland, Samantha Wilkinson, Srinivasa Vittal Katikireddi, Jiafeng Pan, Ting Shi, Steven Kerr, Uktarsh Agrawal, Igor Rudan, Colin R Simpson, Sarah J Stock, John Macleod, Josephine-LK Murray, Colin McCowan, Lewis Ritchie, Mark Woolhouse, Aziz Sheikh

**Affiliations:** 1 Usher Institute, Edinburgh Medical School, University of Edinburgh, Edinburgh, EH16 4UX UK; 2 MRC/CSO Social & Public Health Sciences Unit, University of Glasgow G3 7HR, Glasgow, UK; 3 Department of Mathematics and Statistics, University of Strathclyde, Glasgow, G1 1XH UK; 4 School of Medicine, University of St. Andrews, St Andrews, KY16 9TF UK; 5 School of Health, Wellington Faculty of Health, Victoria University of Wellington, PO Box 600,Wellington 6140 New Zealand; 6 The National Institute for Health Research Applied Research Collaboration West (NIHR ARC West) at University Hospitals Bristol and Weston NHS Foundation Trust, Bristol, BS1 2NT, UK; 7 Public Health Scotland, Glasgow, G2 6QE UK; 8 Academic Primary Care, University of Aberdeen School of Medicine and Dentistry, Aberdeen, AB24 3FX UK

**Keywords:** Population trends, public health, statistics and research methods

## Abstract

**Objectives:**

COVID-19 has resulted in the greatest disruption to National Health Service (NHS) care in its over 70-year history. Building on our previous work, we assessed the ongoing impact of pandemic-related disruption on provision of emergency and elective hospital-based care across Scotland over the first year of the pandemic.

**Design:**

We undertook interrupted time-series analyses to evaluate the impact of ongoing pandemic-related disruption on hospital NHS care provision at national level and across demographics and clinical specialties spanning the period 29 March 2020–28 March 2021.

**Setting:**

Scotland, UK.

**Participants:**

Patients receiving hospital care from NHS Scotland.

**Main outcome measures:**

We used the percentage change of accident and emergency attendances, and emergency and planned hospital admissions during the pandemic compared to the average admission rate for equivalent weeks in 2018–2019.

**Results:**

As restrictions were gradually lifted in Scotland after the first lockdown, hospital-based admissions increased approaching pre-pandemic levels. Subsequent tightening of restrictions in September 2020 were associated with a change in slope of relative weekly admissions rate: –1.98% (–2.38, –1.58) in accident and emergency attendance, –1.36% (–1.68, –1.04) in emergency admissions and –2.31% (–2.95, –1.66) in planned admissions. A similar pattern was seen across sex, socioeconomic status and most age groups, except children (0–14 years) where accident and emergency attendance, and emergency admissions were persistently low over the study period.

**Conclusions:**

We found substantial disruption to urgent and planned inpatient healthcare provision in hospitals across NHS Scotland. There is the need for urgent policy responses to address continuing unmet health needs and to ensure resilience in the context of future pandemics.

## Introduction

Almost three months following the emergence of severe acute respiratory syndrome coronavirus 2 in Wuhan, the World Health Organization (WHO) declared a global coronavirus disease 2 (COVID-19) pandemic on 11 March 2020.^1^ COVID-19 swiftly placed immense pressure on the provision of routine healthcare as the number of people infected with SARS-CoV-2 rapidly increased.^2^ The UK and Scottish Governments responded by introducing national lockdowns on 23 March 2020. Concurrently, many aspects of healthcare provision were curtailed including suspending or cancelling planned surgery and reducing the number of face-to-face clinical assessments.^2^ These actions were taken to focus resources on patients with COVID-19 and to minimise transmission of the virus. We previously investigated the scale of the disruption on the provision of secondary care in Scotland over three months following the initial lockdown (until week ending 28 June 2020) and found that the usage of hospital-based services was severely disrupted.^3^ In addition to directly causing morbidity and mortality, healthcare disruptions represent indirect effects of the COVID-19 pandemic that have likely led to increased morbidity and mortality.^4–6^

**Figure 1.**
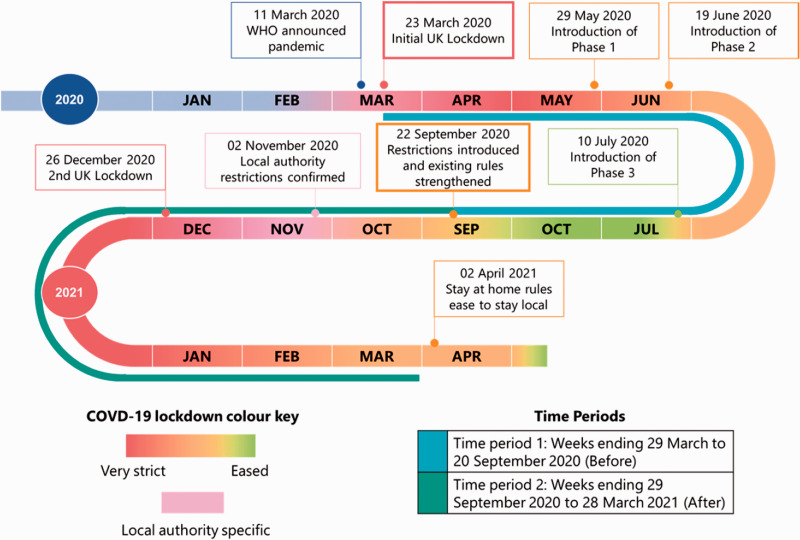
Scotland’s COVID-19 lockdown roadmap from 2020 to April 2021 with the study periods for reference. References found in Table S1.

To manage the pandemic after the imposition of the first UK-wide lockdown, the Scottish Government introduced three phases to allow gradual easing of restrictions (see Figure 1 and Supplementary Table S1 for a timeline and additional details). Briefly, Scotland exited the initial lockdown with stepwise easing of restrictions that started with Phase 1 (most strict) from 29 May 2020, to Phase 3 (least strict) commencing on 9 July 2020. However, cases of COVID-19 started to rise once more in August 2020 and additional measures were introduced on 22 September 2020. This was followed by local authorities entering a tiered system of restrictions based on regional rate of infection commencing on 2 November 2020. Then on 26 December 2020, the whole of Scotland and the UK moved to its second national lockdown, with similar social restrictions to those imposed during the initial lockdown in March 2020.

In this study, we aimed to investigate how the relaxing and tightening of these restrictions impacted on hospital-based care in Scotland over a 12-month period from the imposition of the first lockdown.

## Methods

### Study design and data sources

We extended our initial analysis and undertook an interrupted time-series (ITS) analysis^7,8^ to assess the impact of re-introduced lockdown restrictions on 22 September 2020. We studied two time-periods: (1) Before: weeks ending 28 March 2020 to 27 September 2020; and (2) After: weeks ending 4 October 2020 to 28 March 2021. The timeline of Scotland’s lockdown roadmap illustrates these time-periods and the relevant events that occurred during them (Figure 1, Supplementary Table S1).

We used weekly hospital data in Scotland spanning a timeframe of one year, from the weeks ending 29 March 2020 to 28 March 2021. Data were obtained from the Public Health Scotland R Shiny app ‘Wider impacts of COVID-19’.^9^ To capture healthcare disruption in secondary care, we analysed three outcomes: accident and emergency (A&E) attendances, emergency hospital admissions, and planned hospital admissions across National Health Service (NHS) Scotland. The three outcomes were stratified by demographic variables and clinical specialties), (see Mulholland et al.^3^ for further details on the data sources and outcome definitions). We anticipated little selection bias in these data sources since they routinely captured all hospital-based activity across Scotland.

### Data fields

Outcomes were measured on a weekly basis and were quantified as the relative percentage change of the weekly attendances/admissions to the two-year weekly average of 2018–2019 usage.

We considered healthcare disruption by demographic variables and selected clinical specialties. These data were categorised as follows: sex (male, female); age group (<5, 5–14, 15–44, 45–64, 65–84 and 85+ years) with age-bands in line with our previous work^3^; deprivation (defined using the Scottish Index of Multiple Deprivation [SIMD]^10^ quintiles: 1 (most deprived) to 5 [least deprived]); and clinical specialties for hospital admissions only (A&E, Cancer, Cardiology, Gynaecology, Medical, Paediatrics [medical], Paediatrics [surgical] and Surgery).

### Statistical methods

To compare whether rates differed between the two-year historical average and the 2020–2021 levels, the mean counts were compared at four week-time periods: (1) four weeks before change-point (weeks ending 6 September to 27 September 2020); (2) four weeks after change-point (weeks ending 3 October 3 to 31 October 2020); and (3) four weeks before the end of the study (weeks ending 28 February 2021 to 28 March 2021). Mean counts were compared using two-sample Wilcoxon signed rank tests and using the four counts in each sample.

Modelling was conducted using segmented/piecewise linear regression models containing a linear slope for time, a binary term for the change-point (0: before intervention, 1: after intervention) and an interaction between the two terms. This interaction accounts for any step changes (changes to the intercept) and slope changes (changes to the weekly rate) before and after the intervention. Estimates for these step and slope changes were calculated using the before time period^1^ as the reference group, meaning an estimate of 0 suggested there was no change in the intercept or slope and a positive estimate suggested that there was an increase in the intercept or slope after the change-point. The addition of each of the variables was explored in turn as categorical terms using interactions as outlined in our previous study.^3^

To compare these different models, the Akaike Information Criteria and the Bayesian Information Criterion were used. We checked assumptions of linear regression by assessing the histogram of residuals, the normal QQ plot of residuals and residuals vs. fitted values. We also checked for the presence of autocorrelation using the autocorrelation function and the partial autocorrelation function. To assess the fit of the model parameters, the maximum likelihood ratio test was used. All estimates were reported using 95% confidence intervals (CI).

The analyses were undertaken by RM and independently verified by SAS in R software, version 3.6.1 (http://www.R-project.org). All R code scripts will be made available on the EAVE II GitHub page (https://github.com/EAVE-II/Impact-of-COVID-19-on-secondary-care-in-Scotland) on publication.

### Reporting guideline

We used the Reporting of studies Conducted using Observational Routinely-collected Data (RECORD)^11^ extended from the Strengthening the Reporting of Observational Studies in Epidemiology (STROBE) statement on reporting guidelines to support the communication of findings (Supplementary Table S2).

### Ethical permissions

Ethical approval was not required for this study since the data are aggregated and open sourced on Public Health Scotland (PHS).^9^

### Role of the funding source

The funders had no role in study design, data analysis, decision to publish or preparation of the manuscript.

## Results

**Figure 2.**
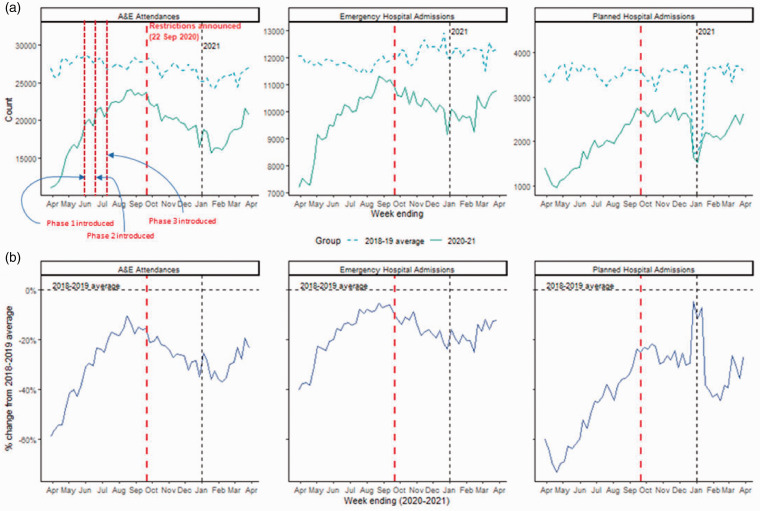
Overall rates of A&E attendances (left), emergency (middle) and planned hospital admissions (right) from weeks ending 29 March 2020 to 28 March 2021. Red vertical dotted line represents the announcement of re-introduced lockdown measures on 22 September 2020 and black dotted line represents the start of 2021. (a) Counts by 2018–2019 average (dotted line) and 2020–2021 (solid line). (b) Relative percentage change of the 2020–2021 counts to the 2018–2019 average, where 0 represents the two-year historical average.

**Figure 3.**
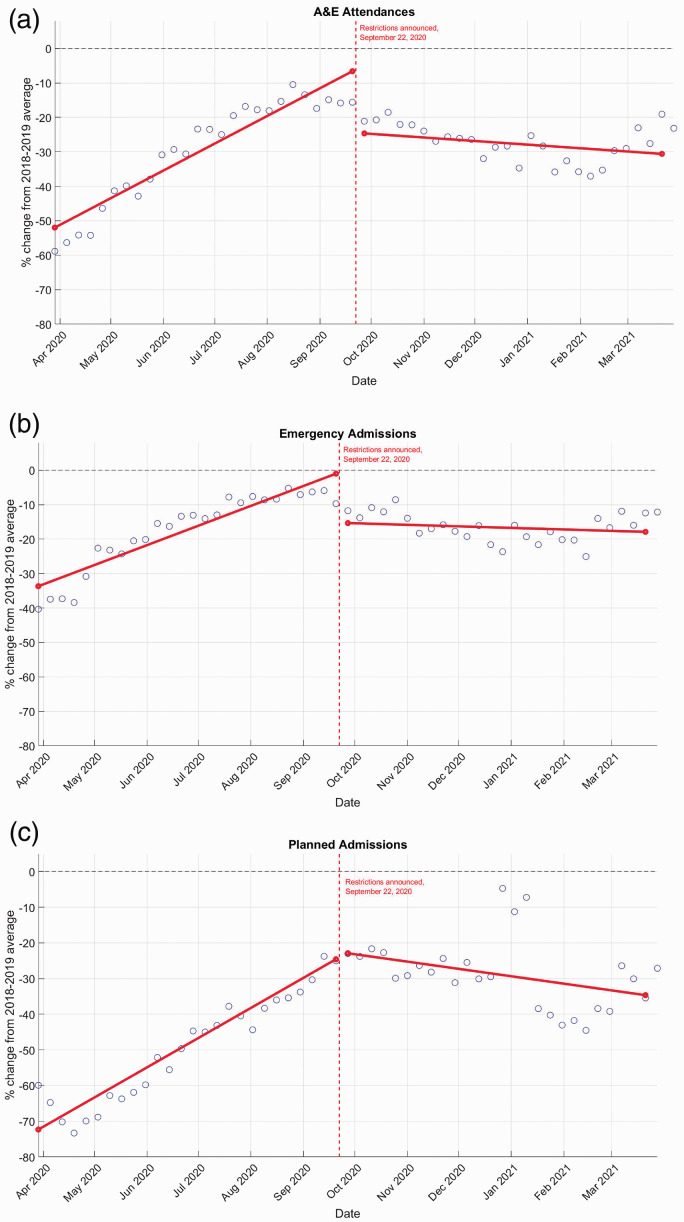
Fitted lines of segmented regression models for A&E attendances, emergency and planned hospital admissions across Scotland. Points represent weekly percentage changes between 2020–2021 and 2018–2019 average for weeks ending 29 March 2020 to 28 March 2021. Vertical line represents change-point (re-introduced lockdown measures announcement 22 September 2020). Horizontal line at 0 is the 2018–2019 average. (a) A&E attendance. (b) Emergency admissions. (c) Planned admissions.

Usage of hospital services steadily increased after the first UK lockdown approaching the two-year historical average as lockdown restrictions were gradually eased in phases (see Figure 1 for the timeline) and peaked during summer 2020. Figure 2 illustrates the count and the percentage change compared to the 2018–2019 average for A&E attendances, and emergency and planned hospital admissions (see Supplementary Tables S3–S5 for two-sample statistical comparison). As restrictions were re-introduced starting 22 September 2020, we observed an immediate decline in A&E attendance and emergency admissions (Figure 3). The introduction of restrictions was associated with a change in level and slope of relative admissions rate: level change of –19.79% (95% CI –25.86, –13.71) in A&E attendance, –15.58% (95% CI –20.50, –10.67) in emergency admissions, –0.16% (95% CI –9.33, 9.01) and in planned admissions; slope change of –1.98% (95% CI –2.38, –1.58) in A&E attendance, –1.36% (95% CI –1.68, –1.04) in emergency admissions and –2.31% (95% CI –2.95, –1.66) in planned admissions (Table 1).

Similar interruption patterns were observed across the demographic characteristics: age, sex and deprivation (Supplementary Figures S1–S3). Age was shown to have the most variability across the different demographic factors, with sex and deprivation not displaying differing patterns amongst their groups (Supplementary Figure S1).

**Figure 4.**
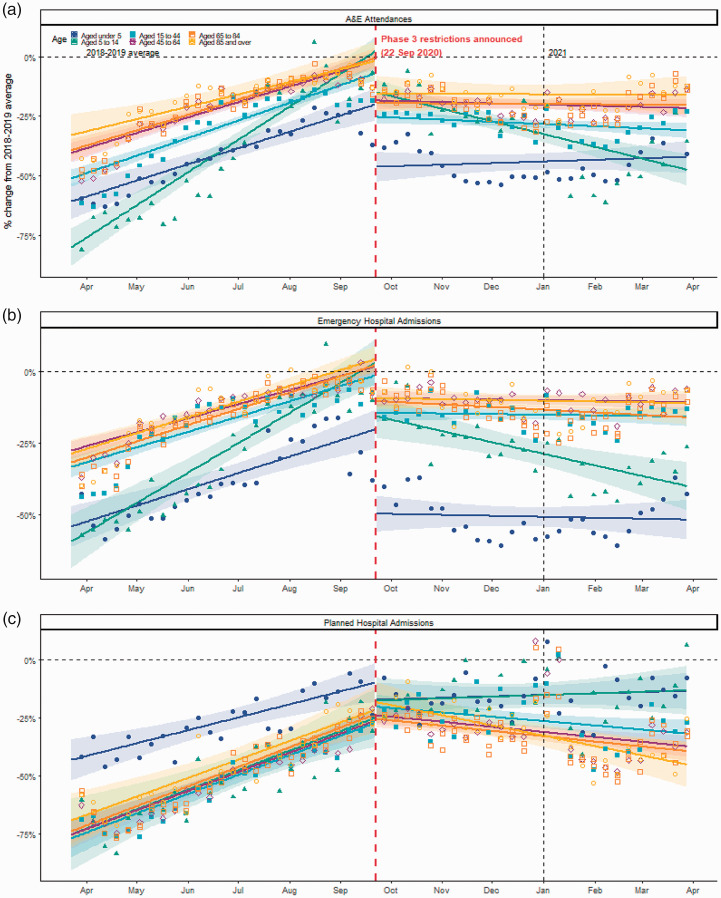
Fitted lines of segmented regression models by age groups for A&E attendances (a) and emergency (b) and planned hospital admissions (c) across Scotland. Points represent weekly percentage changes between 2020–2021 and 2018–2019 averages for weeks ending 29 March 2020 to 28 March 2021. Vertical lines represent change-point (re-introduced lockdown measures announcement 22 September 2020) and the beginning of 2021. Horizontal line is the 2018–2019 average at 0. Shaded areas around lines represent 95% confidence intervals.

 For emergency care (both A&E visits and emergency admissions), those aged under five years were the most impacted by the initial lockdown in March 2020 and this age group continued to have the lowest usage throughout 2020 and 2021 in comparison to the two-year historical average (Figure 4). Furthermore, the re-introduction of restrictions led to the sharpest decline in A&E attendance and admissions in the 5–14-year age group with the slope change dropping by 0.6% per week (0.5–0.7) in A&E attendances and 0.5% per week (0.4–0.6) in emergency admissions (Supplementary Table S6). Trends in the remaining age groups (≥15 years) clustered together and showed a gradual increase after the UK lockdown until the re-introduced lockdown measures, where levels remained steadily below historic levels (Figure 4, Supplementary Tables S3, S4 and S6).

For emergency hospital admissions, the clinical areas exhibiting a continual increase towards pre-pandemic levels were those associated with A&E, cancer and cardiology, all of which in the last four weeks of the study either superseded or were not substantially different from the two-year historical average (Supplementary Figure S5 and Table S4). Surgical paediatrics increased sharply after the initial lockdown, peaked at the time of the re-introduced measures where levels exceeded the two-year average and fell abruptly towards similar levels to the historic average (Supplementary Figure S5 and Table S4). The remaining clinical areas remained well below the previous levels, with medical paediatrics having the lowest levels in comparison to the previous levels (Supplementary Figure S5).

Planned hospital admissions for cancer and medical paediatrics showed minimal reduction after the re-introduced measures, where levels continued to increase and reached similar levels to the two-year historic average by the end of the study (Supplementary Figure S5 and Table S5). Planned hospital admissions for the remaining specialties showed a reduction after the re-introduction of restrictions and maintained levels below historic levels during the follow-up (Supplementary Figure S5).

## Discussion

Hospital healthcare provision remained enormously disrupted across Scotland 12 months after the imposition of the first national lockdown with these impacts being seen across sex, age groups, deprivation groups and most clinical specialties. An overall pattern comprising three key trends emerged: first, there was an immediate and substantial reduction in numbers attending hospital starting 2–3 weeks preceding the announcement of the first UK lockdown^3^; second, recovery commenced during lockdown from mid-April 2020 until September 2020 with rates of healthcare utilisation slowly approaching pre-pandemic levels as restrictions were gradually lifted; and third, the numbers attending hospital started to decrease again following the re-imposition of restrictions on 22 September 2020 that continued throughout the remaining of the study period up to March 2021. Furthermore, despite recovery, hospital-based activity remained at well-below levels in preceding years, even when COVID-19 restrictions were most relaxed during Phase 3 from July to early September 2020. Compared to other age groups, the recovery of emergency hospital usage for children (under 5 and 5–14 years) was the lowest compared to historic levels. However, the same age groups showed the most recovery in planned hospital usage.

To our knowledge, this is the first national-level study that assessed the one-year impact of the pandemic on elective and emergency hospital usage. The key strengths of this analysis include covering the entire population, the length of follow-up (one year) and the use of routinely collected clinician-recorded data. Furthermore, the ITS design employed is a powerful methodological tool to investigate the impact of an intervention on the performance of a healthcare system, particularly when that intervention is unforeseen or there is limited control over the time-point of the intervention.^12^

While an ITS analysis can overcome some of the biases inherent in observational data, challenges remain in inferring causality. A key challenge with an ITS analysis is selecting the exact time-point of the intervention. Where there is a clear event or intervention, it is easy to identify what would be the pre- and post-intervention data points. In this study, however, we sought to adopt a time-point associated with a healthcare policy measure (easing and tightening of restrictions) in response to the prevalence of COVID-19 in Scotland. The timeline of the COVID-19 pandemic (Supplementary Table S1) suggested two interventions: the ‘eat-out-to-help-out’ scheme, which started on 3 August 2020, and the tightening of restrictions, which started on 22 September 2020, as visual examination of hospital activity (Supplementary Figure S1) revealed a downward trend starting during Phase 3. Preliminary analyses showed that both time-points produced similar results, but we considered 22 September 2020 a more appropriate time-point as planned hospital admissions were still increasing throughout August and September 2020.

COVID-19 has had a very major disruption in hospital services in the UK, particularly around the time lockdown was first imposed. Early reporting on 6 April 2020 revealed a 49% decrease in activity at emergency departments in England in the week after lockdown commenced compared to the last week of February 2020.^13^ Similar magnitudes of change have emerged from subsequent ITS analyses of national data, with attendances down 41% for A&E, 26% for emergency hospital admissions and 61% for planned hospital admissions across Scotland,^3^ and 51% for emergency departments at hospitals in England,^14^ compared to preceding years. Explanations for the reduction in hospital visits may include population behavioural changes related to the fear of contracting COVID-19 or reluctance to access healthcare to avoid overwhelming the system, a reduction in other seasonal illnesses^15^ due to less social interactions, a reduction in accidents due to less vehicular use^13^ and cancellation of routine hospital services to improve pandemic response capacity.

As the pandemic progressed, a growing number of UK studies have assessed the pattern of disruption to hospital services over longer timescales. Studies largely concur that a recovery in hospital services gradually commenced after the first lockdown in March 2020, as observed with overall attendances at emergency departments in England (based on data until June 2020),^14^ hospital visits for specific conditions such as acute coronary syndromes,^16^ number of operations for certain cancers (colorectal, colon and rectum cancer) in England^17^ and surgical activity in England and Wales (based on data until September 2020).^18^ Recovery of hospital services was halted with the tightening of restrictions in September 2020. However, the decline was not as steep compared to that observed with the instigation of the initial lockdown. Similarly, a gradual decline in hospital surgical activity in England and Wales was also observed from September 2020.^18^ This relatively minor disruption could be due to a combination of factors. During the pandemic, there was increased public messaging to make the population aware that in an emergency individuals should seek medical help.^19^ In addition, the NHS has developed new processes in response to the COVID-19 pandemic and as a result more stringent infection control measures are now in place along with the allocation of resources and intent to resume and continue routine healthcare,^20^ thereby allowing for resumption of many hospital services. Besides the UK, reductions in planned hospital admission have also been observed in other countries including Japan,^21^ Belgium,^22^ Hong Kong,^23^ Norway,^24^ Sierra Leone^25^ and South Korea.^26^

The severe disruptive effect of COVID-19 to emergency paediatric care in hospitals in Scotland was also observed in England, with greater reductions in children attending emergency departments compared to other age groups.^14^ Before the pandemic, there was generally a high usage of hospital emergency departments for paediatric services. A study of a national dataset across England showed that children accounted for 21% of attendances to A&E (0–15 years of age).^27^ Moreover, non-urgent use of A&E for paediatric illness is considered high, on average accounting for 41.06% (±15.16%) of presentations at A&E,^28^ with the majority of the non-urgent attendances occurring for children aged 0–4 years.^29^ These non-urgent attendances are considered to be largely driven by parental behaviour: an amplified concern about an illness that is perceived to be serious, need for reassurance from paediatric specialists, lack of awareness of other health service options (e.g. NHS 111, out-of-hours primary care), influence of parental social network and a low confidence in illness assessment by the parents.^30^ COVID-19 may have altered aspects of this parental behaviour, resulting in a reduction in seeking non-urgent paediatric care from emergency hospital services. As the pandemic continues, these changes in parental behaviour may be persisting, since emergency paediatric care remained below historic levels and a reduction in the usage of emergency paediatric care was once again observed as restrictions tightened from September 2020 in Scotland.

The substantial recovery during and post lockdown demonstrates that the system has the ability to adapt in an evolving global health crisis. This has occurred alongside a growing knowledge of the biology, pathogenesis and epidemiology of the virus and disease, the development of treatments and vaccines for the virus, and the reorganisation of healthcare in a changing environment. However, the effects of the disruption of health services are likely long-lasting, particularly with regard to the implications of individuals not receiving appropriate routine healthcare. This is likely to have increased morbidity and possibly mortality in the population.^4,5^ There is also the impact of postponing planned hospital admissions on the increased risk of patient morbidity and on quality of life.^6^ If reductions in hospital activity were primarily due to fears of contracting SARS-CoV-2 in a hospital setting, then these concerns may have been largely mitigated through the public health messaging to reassure the population that they should still seek hospital treatment during the pandemic.^19^ However, as the pandemic has evolved, hospital attendances continue to remain well below historic levels; thus, it is important to determine if the continued disruption is due to individuals not seeking care for non-urgent ailments or if there are other reasons contributing to this. It is important to try and disentangle avoidable morbidity and non-urgent emergency hospital attendances. In addition, continued monitoring of the levels of hospital activity can provide crucial information on impact of the changing COVID-19 pandemic. Lastly, there is a need to introduce preventive measures in hospitals to protect patients, healthcare workers and the public. With the COVID-19 pandemic evolving into an endemic, such measures should instil public confidence and encourage them to seek healthcare, if needed.

In conclusion, the COVID-19 pandemic has had a major, persistent and disruptive impact on hospital service provision across Scotland. Despite the easing of restrictions and some recovery in the usage of hospital-based care, activity remained well below historic levels with likely major consequences for avoidable morbidity and possibly mortality.

**Table 1.** Level and slope before the change-point, and the change in level and slope after the change-point for A&E attendance, emergency and planned hospital admissions with 95% confidence intervals.

	A&E attendance	Emergency hospital admissions	Planned hospital admissions
Level before change-point (95% CI)	–51.96 (–56.10, –47.83)	–33.67 (–37.02, –30.32)	–72.31 (–78.99, –65.64)
Slope before change-point (95% CI)	1.75 (1.47, 2.01)	1.26 (1.04, 1.48)	1.84 (1.40, 2.27)
Level change after change-point (95% CI)	–19.79 (–25.86, –13.71)	–15.58 (–20.50, –10.67)	–0.16 (–9.33, 9.01)
Slope change after change-point (95% CI)	–1.98 (–2.38, –1.58)	–1.36 (–1.68, –1.04)	–2.31 (–2.95, –1.66)

Note: All measures are in % change compared to the 2018–2019 mean.

## Supplemental Material

sj-pdf-1-jrs-10.1177_01410768221095239 - Supplemental material for Impact on emergency and elective hospital-based care in Scotland over the first 12 months of the pandemic: interrupted time-series analysis of national lockdowns Click here for additional data file. Supplemental material, sj-pdf-1-jrs-10.1177_01410768221095239 for Impact on emergency and elective hospital-based care in Scotland over the first 12 months of the pandemic: interrupted time-series analysis of national lockdowns by Syed Ahmar Shah, Rachel H Mulholland, Samantha Wilkinson, Srinivasa Vittal Katikireddi, Jiafeng Pan, Ting Shi, Steven Kerr, Uktarsh Agrawal, Igor Rudan, Colin R Simpson, Sarah J Stock, John Macleod, Josephine-LK Murray, Colin McCowan, Lewis Ritchie, Mark Woolhouse and Aziz Sheikh in Royal Society of Medicine

## Declarations

**Competing Interests:** The author(s) declared the following potential conflicts of interest with respect to the research, authorship, and/or publication of this article: AS is a member of the Scottish Government Chief Medical Officer’s COVID-19 Advisory Group and its Scottish Government’s Standing Committee on Pandemics, and the UK Government’s New and Emerging Respiratory Virus Threats (NERVTAG) Risk Stratification Subgroup. SVK was co-chair of the Scottish Government’s Expert Reference Group on Ethnicity and COVID-19 and a member of the UK Scientific Advisory Group on Emergencies (SAGE) subgroup on ethnicity. All authors declare no potential conflicts of interests with respect to the research, authorship, and/or publication of this article.

**Funding:** The author(s) disclosed receipt of the following financial support for the research, authorship, and/or publication of this article: This analysis is part of the Early Pandemic Evaluation and Enhanced Surveillance of COVID-19 (EAVE II) study. EAVE II is funded by the Medical Research Council (MR/R008345/1) with the support of BREATHE – The Health Data Research Hub for Respiratory Health (MC_PC_19004), which is funded through the UK Research and Innovation Industrial Strategy Challenge Fund and delivered through Health Data Research UK. Additional support has been provided through the Scottish Government DG Health and Social Care. SAS and AS are also supported by the COVID-19 Longitudinal Health and Wellbeing National Core Study, funded by the Medical Research Council (MC_PC_20030). SVK acknowledges funding from a NRS Senior Clinical Fellowship (SCAF/15/02), the Medical Research Council (MC_UU_00022/2) and the Scottish Government Chief Scientist Office (SPHSU17). JM is partly funded by the National Institute for Health Research Applied Research Collaboration West (NIHR ARC West).

**Ethics approval:** Not applicable.

**Guarantor:** SAS.

**Contributorship:** AS conceived this manuscript. SW, SAS and RHM led the writing and analysis of this manuscript. All authors contributed to drafting the paper and revised the manuscript for important intellectual content. All authors had final responsibility for the decision to submit for publication. SAS accepts full responsibility for the work.

**Acknowledgements:** We are grateful for the Public Health Scotland team who have created and monitor the dashboard for the underlying data.

**Data accessibility:** Not commissioned; peer-reviewed by Francesco Chirico, Dheeraj Sharma and Julie Morris.

**Provenance:** Data is publicly available on the PHS COVID-19 wider impacts dashboard (https://scotland.shinyapps.io/phs-covid-wider-impact/).

**ORCID iDs:** Syed Ahmar Shah https://orcid.org/0000-0001-5672-0443 Ting Shi https://orcid.org/0000-0002-4101-4535 Uktarsh Agrawal https://orcid.org/0000-0001-5181-6120

**Supplemental Material:** Supplemental material for this article is available online.
