# Supplementary material for: Impact on emergency and elective hospital-based care in Scotland over the first 12 months of the pandemic: interrupted time-series analysis of national lockdowns
Source: J R Soc Med. 2022 May 3;115(11):429–38. doi: 10.1177/01410768221095239 (PMC9723811; doi:10.1177/01410768221095239)
Supplement: sj-pdf-1-jrs-10.1177_01410768221095239 - Supplemental material for Impact on emergency and elective hospital-based care in Scotland over the first 12 months of the pandemic: interrupted time-series analysis of national lockdowns [file sj-pdf-1-jrs-10.1177_01410768221095239.pdf]

Supplementary Material

Figures

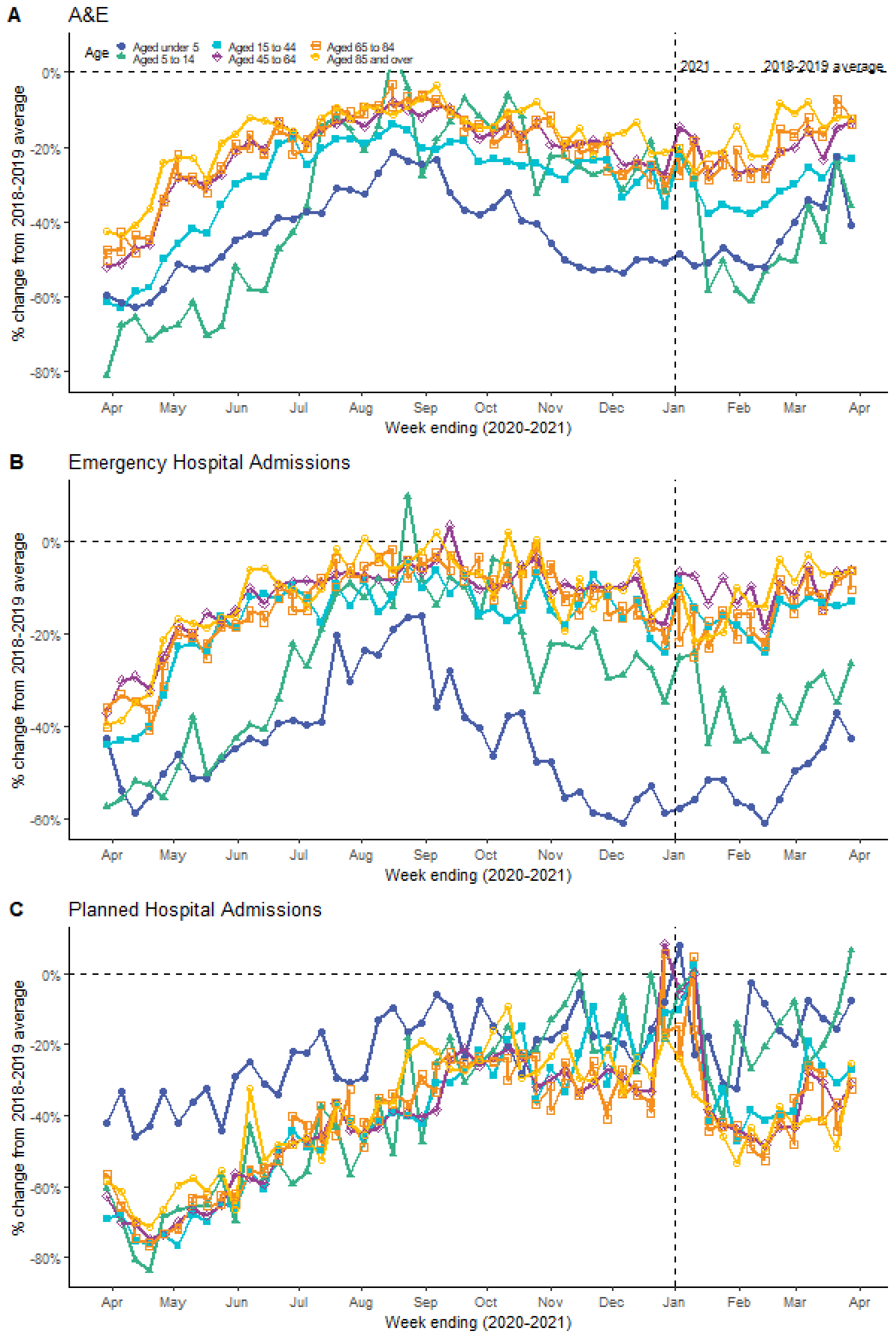

Figure S1: Trends of % changes to the 2018-2019 average for A&E Attendances (A), emergency (B) and planned hospital admissions (C) by age groups.

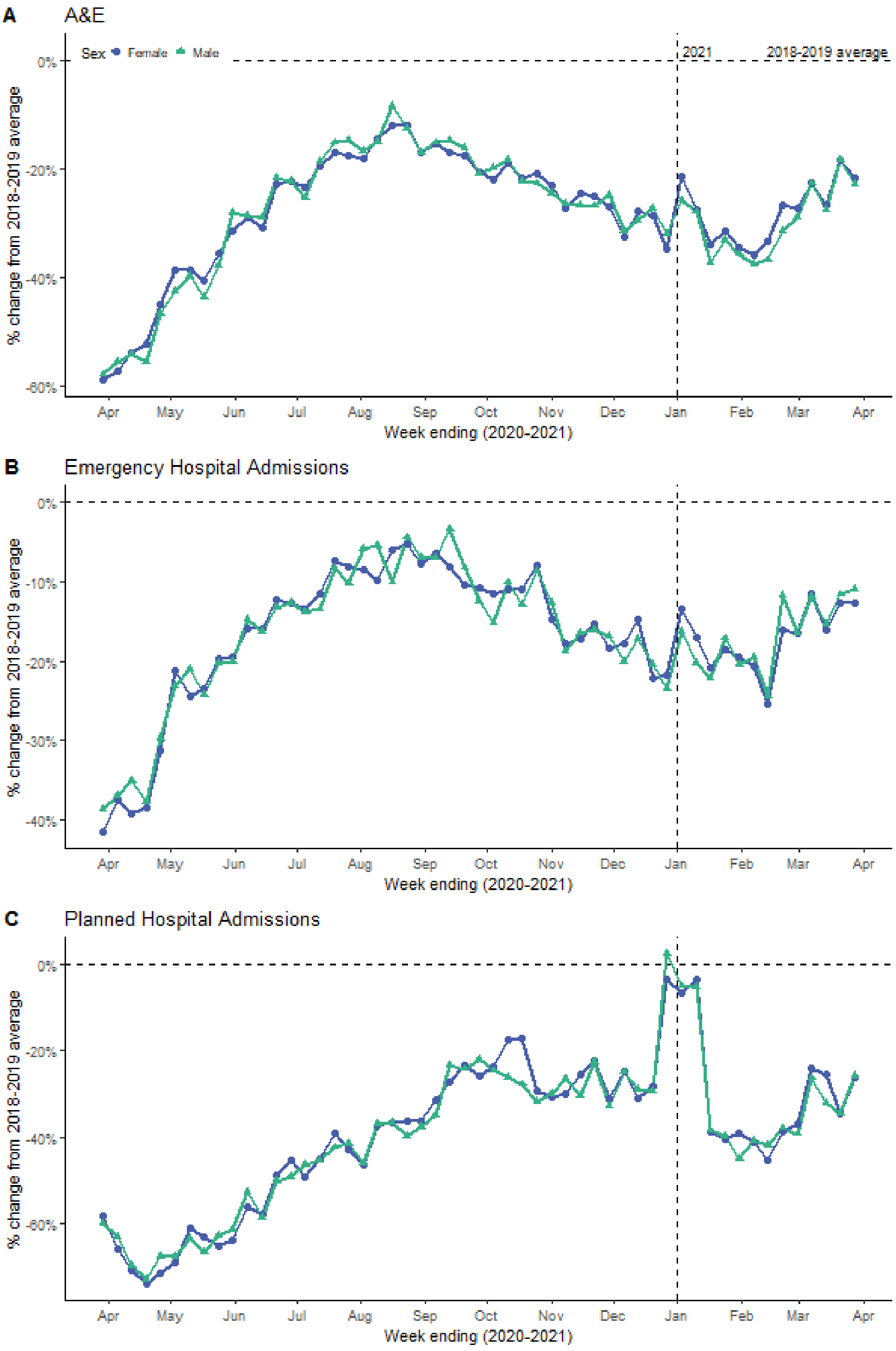

Figure S2: Trends of % changes to the 2018-2019 average for A&E Attendances (A), emergency (B) and planned hospital admissions (C) by sex.

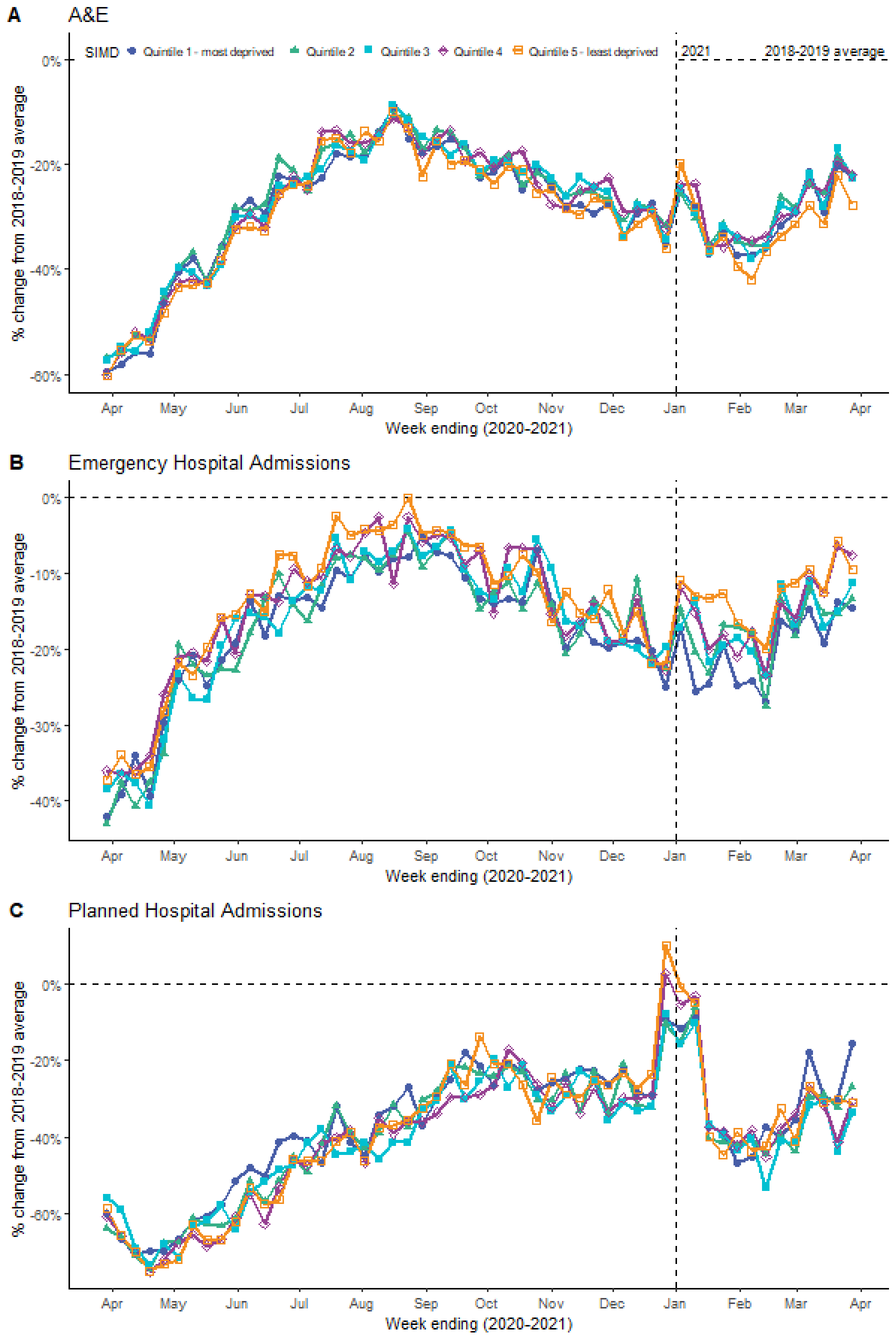

Figure S3: Trends of % changes to the 2018-2019 average for A&E Attendances (A), emergency (B) and planned hospital admissions (C) by deprivation quintiles (via SIMD).

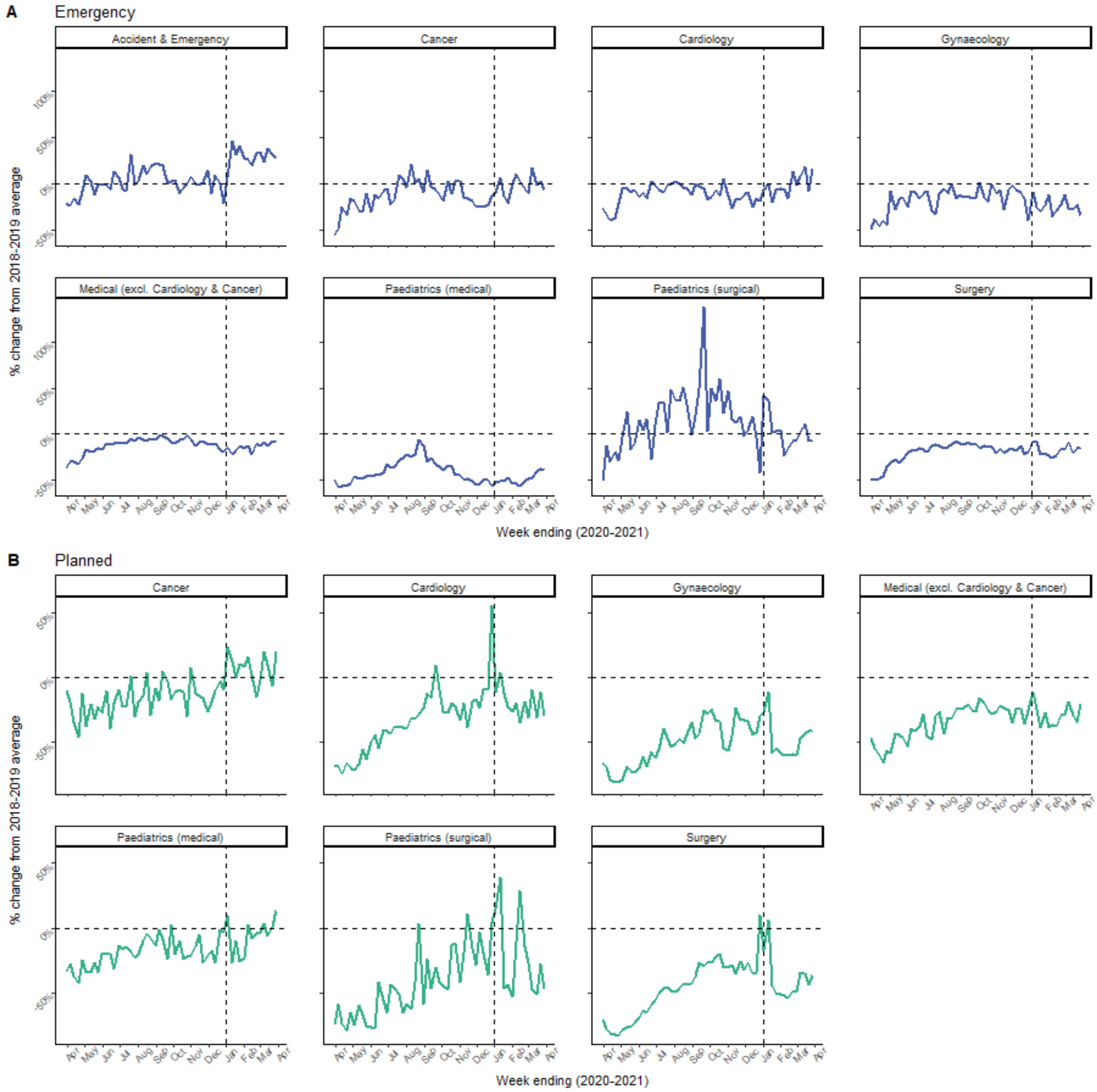

Figure S4: Trends of % changes to the 2018-2019 average for A&E Attendances (A), emergency (B) and planned hospital admissions (C) by clinical specialties.

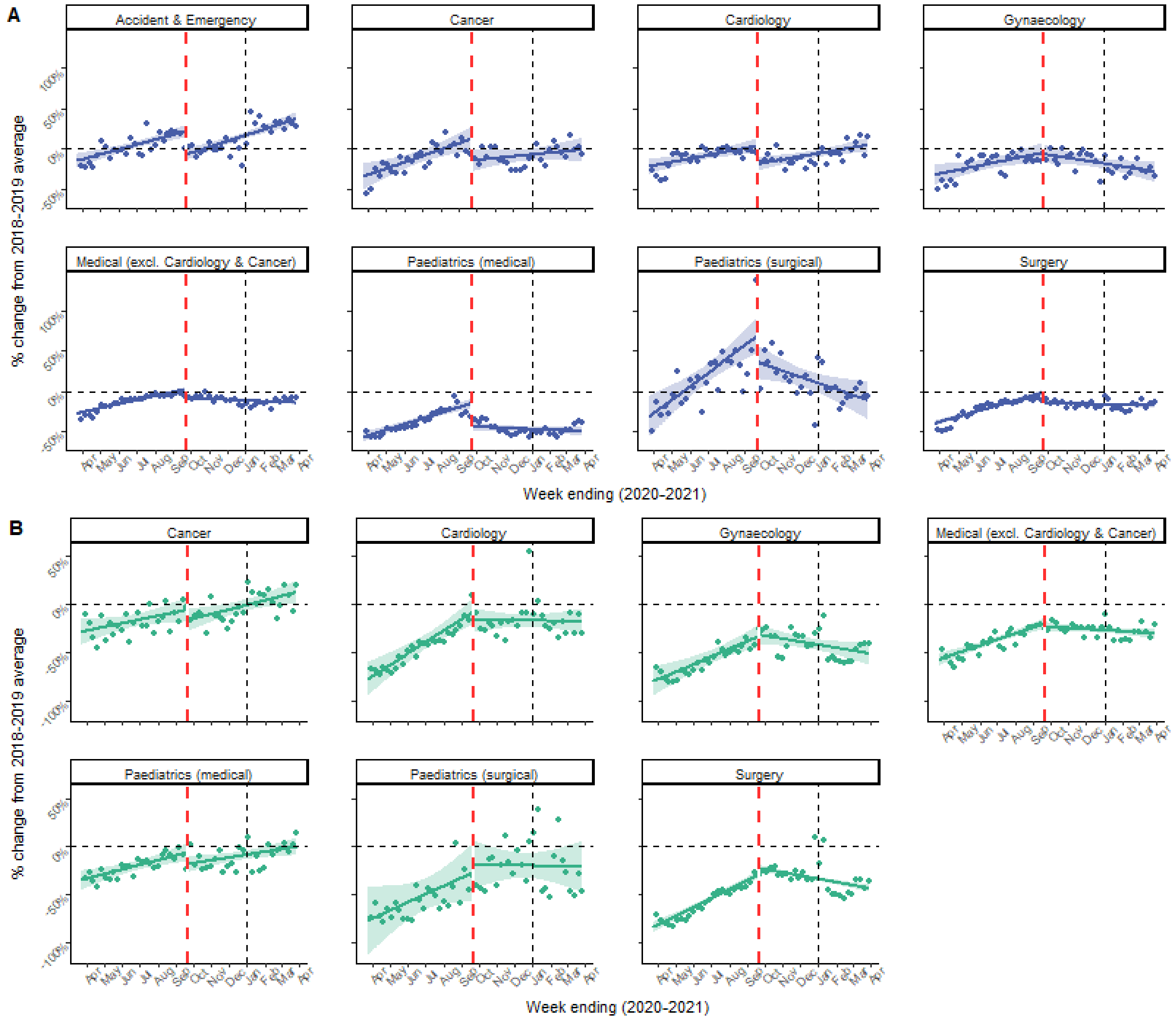

Figure S5: Fitted lines of segmented regression models by clinical specialties for emergency (A) and planned hospital admissions (B) across Scotland. Points represent weekly percentage changes between 2020-21 and 2018–2019 average for weeks ending 29 March 2020 to 28 March 2021. Vertical lines represent change-point (reintroduced lockdown measures announcement 22 September 2020) and the beginning of 2021. Horizontal line is the 2018–2019 average at 0. Shaded areas round lines represent 95% confidence intervals.

Tables

Table S1: Scotland COVID-19 pandemic lockdown roadmap of main events

Announcement date	Commencement date	Announcement	URL
23-Mar-20	23-Mar-20	UK Lockdown	https://www.gov.scot/publications/first-minister-covid-19-update-1/
28-May-20	29-May-20	Lockdown begins to ease in Phase 1	https://www.gov.scot/news/lockdown-begins-to-ease-in-phase-1/
18-Jun-20	19-Jun-20	Introduction of Phase 2	https://www.gov.scot/news/gradual-introduction-of-phase-2/
09-Jul-20	10-Jul-20	Phase 3: Scotland's route map update	https://www.gov.scot/publications/coronavirus-covid-19-framework-decision-making-scotlands-route-map-through-out-crisis-phase-3-update/
13-Jul-20	03-Aug-20	Eat out to help out introduced	https://www.visitscotland.org/news/2020/eat-out-to-help-out
01-Oct-20	22-Sep-20	Phase 3: Further restrictions introduced and existing rules strengthened	https://www.gov.scot/publications/coronavirus-covid-19-phase-3-scotlands-route-map-update-1-october-2020/
29-Oct-20	02-Nov-20	Local authority levels confirmed	https://www.gov.scot/news/local-authority-levels-confirmed/
19-Dec-20	25-Dec-20	Christmas easing	https://www.gov.scot/news/new-guidance-issued-for-the-festive-period/
19-Dec-20	26-Dec-20	Level 4 restrictions applied	https://www.gov.scot/news/new-guidance-issued-for-the-festive-period/
30-Mar-21	02-Apr-21	Stay at home eased to stay local	https://www.gov.scot/news/stay-local-from-2-april/

Table S2: The RECORD statement – checklist of items, extended from the STROBE statement, that should be reported in observational studies using routinely collected health data.

	Item No.	STROBE items	Location in manuscript where items are reported	RECORD items	Location in manuscript where items are reported
**Title and abstract**					
	1	(a) Indicate the study's design with a commonly used term in the title or the abstract (b) Provide in the abstract an informative and balanced summary of what was done and what was found	Pages 1 and 2	RECORD 1.1: The type of data used should be specified in the title or abstract. When possible, the name of the databases used should be included.<br><br>RECORD 1.2: If applicable, the geographic region and timeframe within which the study took place should be reported in the title or abstract.<br><br>RECORD 1.3: If linkage between databases was conducted for the study, this should be clearly stated in the title or abstract.	Pages 1 and 2
**Introduction**					
Background rationale	2	Explain the scientific background and rationale for the investigation being reported	Page 5		
Objectives	3	State specific objectives, including any prespecified hypotheses	Page 5		
**Methods**					
Study Design	4	Present key elements of study design early in the paper	Page 6		
Setting	5	Describe the setting, locations, and relevant dates, including periods of recruitment, exposure, follow-up, and data collection	Page 6		
Participants	6	*(a) Cohort study* - Give the eligibility criteria, and the sources and methods of selection of participants. Describe methods of follow-up<br><br>*Case-control study* - Give the eligibility criteria, and the sources and methods of case ascertainment and control selection. Give the rationale for the choice of cases and controls<br><br>*Cross-sectional study* - Give the eligibility criteria, and the sources and methods of selection of participants	Not applicable	RECORD 6.1: The methods of study population selection (such as codes or algorithms used to identify subjects) should be listed in detail. If this is not possible, an explanation should be provided.<br><br>RECORD 6.2: Any validation studies of the codes or algorithms used to select the population should be referenced. If validation was conducted for this study and not published elsewhere, detailed methods and results should be provided.	Not applicable

		(b) Cohort study - For matched studies, give matching criteria and number of exposed and unexposed<br><br>*Case-control study* - For matched studies, give matching criteria and the number of controls per case		RECORD 6.3: If the study involved linkage of databases, consider use of a flow diagram or other graphical display to demonstrate the data linkage process, including the number of individuals with linked data at each stage.	
Variables	7	Clearly define all outcomes, exposures, predictors, potential confounders, and effect modifiers. Give diagnostic criteria, if applicable.	Pages 6-7	RECORD 7.1: A complete list of codes and algorithms used to classify exposures, outcomes, confounders, and effect modifiers should be provided. If these cannot be reported, an explanation should be provided.	Not in manuscript, but can be found in data source: https://scotland.shinyapps.io/phs-covid-wider-impact/
Data sources/ measurement	8	For each variable of interest, give sources of data and details of methods of assessment (measurement).<br><br>Describe comparability of assessment methods if there is more than one group	Pages 6-7		
Bias	9	Describe any efforts to address potential sources of bias	Page 6		
Study size	10	Explain how the study size was arrived at	Page 6		
Quantitative variables	11	Explain how quantitative variables were handled in the analyses. If applicable, describe which groupings were chosen, and why	Not applicable		
Statistical methods	12	(a) Describe all statistical methods, including those used to control for confounding<br><br>(b) Describe any methods used to examine subgroups and interactions<br><br>(c) Explain how missing data were addressed<br><br>(d) *Cohort study* - If applicable, explain how loss to follow-up was addressed<br><br>*Case-control study* - If applicable, explain how matching of cases and controls was addressed<br><br>*Cross-sectional study* - If applicable, describe analytical methods taking account of sampling strategy<br><br>(e) Describe any sensitivity analyses	Pages 6-8		
Data access and cleaning methods		..		RECORD 12.1: Authors should describe the extent to which the investigators had access to the	Pages 6-7

				database population used to create the study population.<br><br>RECORD 12.2: Authors should provide information on the data cleaning methods used in the study.	
Linkage		..		RECORD 12.3: State whether the study included person-level, institutional-level, or other data linkage across two or more databases. The methods of linkage and methods of linkage quality evaluation should be provided.	Not applicable
**Results**					
Participants	13	(a) Report the numbers of individuals at each stage of the study (*e.g.*, numbers potentially eligible, examined for eligibility, confirmed eligible, included in the study, completing follow-up, and analysed)<br><br>(b) Give reasons for non-participation at each stage.<br><br>(c) Consider use of a flow diagram	Not applicable	RECORD 13.1: Describe in detail the selection of the persons included in the study (*i.e.,* study population selection) including filtering based on data quality, data availability and linkage. The selection of included persons can be described in the text and/or by means of the study flow diagram.	Not applicable
Descriptive data	14	(a) Give characteristics of study participants (*e.g.*, demographic, clinical, social) and information on exposures and potential confounders<br><br>(b) Indicate the number of participants with missing data for each variable of interest<br><br>(c) *Cohort study* - summarise follow-up time (*e.g.*, average and total amount)	Figures 2, Figures S1-S4		
Outcome data	15	*Cohort study* - Report numbers of outcome events or summary measures over time<br><br>*Case-control study* - Report numbers in each exposure category, or summary measures of exposure<br><br>*Cross-sectional study* - Report numbers of outcome events or summary measures	Figures 2, Figures S1-S4		
Main results	16	(a) Give unadjusted estimates and, if applicable, confounder-adjusted estimates and their precision (e.g., 95% confidence interval). Make clear which confounders were adjusted for and why they were included<br><br>(b) Report category boundaries when continuous variables were categorized	Pages 9-10, Figures 3-4, Table 1		

		(c) If relevant, consider translating estimates of relative risk into absolute risk for a meaningful time period			
Other analyses	17	Report other analyses done—e.g., analyses of subgroups and interactions, and sensitivity analyses	Not applicable		
**Discussion**					
Key results	18	Summarise key results with reference to study objectives	Page 11-12		
Limitations	19	Discuss limitations of the study, taking into account sources of potential bias or imprecision. Discuss both direction and magnitude of any potential bias	Page 12	RECORD 19.1: Discuss the implications of using data that were not created or collected to answer the specific research question(s). Include discussion of misclassification bias, unmeasured confounding, missing data, and changing eligibility over time, as they pertain to the study being reported.	Page 13
Interpretation	20	Give a cautious overall interpretation of results considering objectives, limitations, multiplicity of analyses, results from similar studies, and other relevant evidence	Page 11 and 14-15		
Generalisability	21	Discuss the generalisability (external validity) of the study results	Pages 11-13		
**Other Information**					
Funding	22	Give the source of funding and the role of the funders for the present study and, if applicable, for the original study on which the present article is based	Page 15		
Accessibility of protocol, raw data, and programming code		..		RECORD 22.1: Authors should provide information on how to access any supplemental information such as the study protocol, raw data, or programming code.	Page 16

*Reference: Benchimol EI, Smeeth L, Guttmann A, Harron K, Moher D, Petersen I, Sørensen HT, von Elm E, Langan SM, the RECORD Working Committee. The REporting of studies Conducted using Observational Routinely-collected health Data (RECORD) Statement. *PLoS Medicine* 2015; in press.

*Checklist is protected under Creative Commons Attribution (CC BY) license.

Table S3: A&E Attendances - mean differences between 2018-2019 average and 2020-201 for 4 weekly time periods of 4 weeks before change-point (weeks ending 9 September to 27 September 2020), 4 weeks after change-point (weeks ending 4 October to 1 November 2020) and 4 weeks before end date (weeks ending 28 February to 28 March 2021). Stratified by the different characteristics.

**Characteristic**	**Level**	**4 week period**	**2018-2019 Average Mean**	**2020-2021 Mean**	**Difference (p-value)***
Total		4 weeks before change-point	27979.4	23260.5	-4718.9 (p=0.029)
		4 weeks after change-point	26677.5	21118.5	-5559 (p=0.029)
		4 weeks before end date	26142.5	20076.5	-6066 (p=0.029)
Age	Aged under 5	4 weeks before change-point	2511.6	1691.5	-820.1 (p=0.029)
		4 weeks after change-point	2532.2	1594.2	-938 (p=0.029)
		4 weeks before end date	2484	1653.2	-830.8 (p=0.029)
	Aged 5 to 14	4 weeks before change-point	3040.1	2656.2	-383.9 (p=0.029)
		4 weeks after change-point	2386.6	1993.5	-393.1 (p=0.245)
		4 weeks before end date	2808.1	1829	-979.1 (p=0.029)
	Aged 15 to 44	4 weeks before change-point	9828.1	7818.5	-2009.6 (p=0.029)
		4 weeks after change-point	9420.9	7151.5	-2269.4 (p=0.029)
		4 weeks before end date	9141.4	6872.2	-2269.2 (p=0.029)
	Aged 45 to 64	4 weeks before change-point	5906.8	5182	-724.8 (p=0.029)
		4 weeks after change-point	5776.6	4886.5	-890.1 (p=0.029)
		4 weeks before end date	5564.5	4625.2	-939.3 (p=0.029)
	Aged 65 to 84	4 weeks before change-point	2365.5	2084.8	-280.7 (p=0.005)
		4 weeks after change-point	2348.9	1977.6	-371.3 (p=<0.001)
		4 weeks before end date	2223.4	1930.9	-292.5 (p=0.003)
	Aged 85 and over	4 weeks before change-point	1246.2	1114	-132.2 (p=0.029)
		4 weeks after change-point	1284.8	1145.8	-139 (p=0.029)
		4 weeks before end date	1212.2	1069	-143.2 (p=0.029)
Sex	Female	4 weeks before change-point	13296.8	10981.5	-2315.3 (p=0.029)
		4 weeks after change-point	12838.6	10191	-2647.6 (p=0.029)
		4 weeks before end date	12616.4	9826.5	-2789.9 (p=0.029)
	Male	4 weeks before change-point	13967.1	11650.2	-2316.9 (p=0.029)
		4 weeks after change-point	13260.4	10535.8	-2724.6 (p=0.029)
		4 weeks before end date	13040.6	10084	-2956.6 (p=0.029)
SIMD	Quintile 1 - most deprived	4 weeks before change-point	7840.8	6458.8	-1382 (p=0.029)
		4 weeks after change-point	7498	5891	-1607 (p=0.029)
		4 weeks before end date	7461.5	5729	-1732.5 (p=0.029)
	Quintile 2	4 weeks before change-point	6149.9	5131.8	-1018.1 (p=0.029)
		4 weeks after change-point	5973.4	4731.5	-1241.9 (p=0.029)
		4 weeks before end date	5875.9	4557.5	-1318.4 (p=0.029)
	Quintile 3	4 weeks before change-point	4854.1	3988.2	-865.9 (p=0.029)
		4 weeks after change-point	4691.4	3749.5	-941.9 (p=0.029)
		4 weeks before end date	4554.5	3537	-1017.5 (p=0.029)
	Quintile 4	4 weeks before change-point	4328.1	3621.8	-706.3 (p=0.029)
		4 weeks after change-point	4049	3239.8	-809.2 (p=0.029)
		4 weeks before end date	4025.6	3118.2	-907.4 (p=0.029)
	Quintile 5 - least deprived	4 weeks before change-point	3710.1	3006.8	-703.3 (p=0.029)
		4 weeks after change-point	3507	2715.8	-791.2 (p=0.029)
		4 weeks before end date	3481	2538.8	-942.2 (p=0.029)
*Difference column colours refer to whether the means were statistically higher (green), lower (red) or not different (yellow).					

Table S4: Emergency hospital admissions - mean differences between 2018-2019 average and 2020-201 for 4 weekly time periods of 4 weeks before change-point (weeks ending 9 September to 27 September 2020), 4 weeks after change-point (weeks ending 4 October to 1 November 2020) and 4 weeks before end date (weeks ending 28 February to 28 March 2021). Stratified by the different characteristics.

Characteristic	Level	4 week period	2018-2019 Average Mean	2020-2021 Mean	Difference (p-value)*
Total		4 weeks before change-point	11949.9	10942.2	-1007.7 (p=0.029)
		4 weeks after change-point	11975.6	10616.2	-1359.4 (p=0.029)
		4 weeks before end date	12135.9	10540.2	-1595.7 (p=0.029)
Age	Aged under 5	4 weeks before change-point	842.8	542.8	-300 (p=0.029)
		4 weeks after change-point	929.8	536.8	-393 (p=0.029)
		4 weeks before end date	926.2	528	-398.2 (p=0.029)
	Aged 5 to 14	4 weeks before change-point	481.5	423.8	-57.7 (p=0.029)
		4 weeks after change-point	403.8	344.8	-59 (p=0.343)
		4 weeks before end date	501.6	350	-151.6 (p=0.029)
	Aged 15 to 44	4 weeks before change-point	2515	2256.2	-258.8 (p=0.029)
		4 weeks after change-point	2433.5	2104	-329.5 (p=0.029)
		4 weeks before end date	2467.8	2140.5	-327.3 (p=0.029)
	Aged 45 to 64	4 weeks before change-point	2818.1	2685.5	-132.6 (p=0.114)
		4 weeks after change-point	2827.1	2625	-202.1 (p=0.029)
		4 weeks before end date	2870.8	2631	-239.8 (p=0.057)
	Aged 65 to 84	4 weeks before change-point	1974.1	1871.8	-102.3 (p=0.083)
		4 weeks after change-point	2002.4	1844.6	-157.8 (p=0.021)
		4 weeks before end date	2018.7	1819.4	-199.3 (p=0.001)
	Aged 85 and over	4 weeks before change-point	1344.2	1290.2	-54 (p=0.343)
		4 weeks after change-point	1376.6	1315.8	-60.8 (p=0.343)
		4 weeks before end date	1332.1	1252	-80.1 (p=0.114)
Sex	Female	4 weeks before change-point	5941.1	5410.2	-530.9 (p=0.029)
		4 weeks after change-point	5947.6	5329.2	-618.4 (p=0.029)
		4 weeks before end date	6070.6	5264.5	-806.1 (p=0.029)
	Male	4 weeks before change-point	5827.4	5373.2	-454.2 (p=0.029)
		4 weeks after change-point	5864.1	5177.8	-686.3 (p=0.029)
		4 weeks before end date	5966.9	5221	-745.9 (p=0.029)
SIMD	Quintile 1 - most deprived	4 weeks before change-point	3224.1	2911.2	-312.9 (p=0.029)
		4 weeks after change-point	3214.5	2826.5	-388 (p=0.029)
		4 weeks before end date	3270	2760.2	-509.8 (p=0.029)
	Quintile 2	4 weeks before change-point	2734.8	2491	-243.8 (p=0.029)
		4 weeks after change-point	2750	2406.5	-343.5 (p=0.029)
		4 weeks before end date	2804.9	2410.2	-394.7 (p=0.029)
	Quintile 3	4 weeks before change-point	2267.8	2081.5	-186.3 (p=0.029)
		4 weeks after change-point	2325.4	2087.8	-237.6 (p=0.029)
		4 weeks before end date	2370.9	2046.2	-324.7 (p=0.029)
	Quintile 4	4 weeks before change-point	1952.8	1825.2	-127.6 (p=0.029)
		4 weeks after change-point	1958.2	1783.8	-174.4 (p=0.029)
		4 weeks before end date	1985.2	1801.2	-184 (p=0.029)
	Quintile 5 - least deprived	4 weeks before change-point	1624.5	1535	-89.5 (p=0.029)
		4 weeks after change-point	1613	1454.5	-158.5 (p=0.029)
		4 weeks before end date	1634.1	1481.2	-152.9 (p=0.029)
Specialty	Accident & Emergency	4 weeks before change-point	405	447	42 (p=0.081)
		4 weeks after change-point	387.5	377.2	-10.3 (p=0.486)
		4 weeks before end date	289.6	377	87.4 (p=0.029)
	Cancer	4 weeks before change-point	132.4	129.5	-2.9 (p=0.686)
		4 weeks after change-point	133.4	124.8	-8.6 (p=0.686)
		4 weeks before end date	115.4	118.2	2.8 (p=0.663)
	Cardiology	4 weeks before change-point	327.2	304.5	-22.7 (p=0.2)
		4 weeks after change-point	316.4	292.8	-23.6 (p=0.2)
		4 weeks before end date	316.8	342	25.2 (p=0.2)

Characteristic	Level	4 week period	2018-2019 Average Mean	2020-2021 Mean	Difference (p-value)*
	Community	4 weeks before change-point	182.6	136.8	-45.8 (p=0.029)
		4 weeks after change-point	180.4	124.2	-56.2 (p=0.029)
		4 weeks before end date	190.4	127	-63.4 (p=0.029)
	Gynaecology	4 weeks before change-point	204.2	174	-30.2 (p=0.029)
		4 weeks after change-point	185.9	170.5	-15.4 (p=0.381)
		4 weeks before end date	223.6	161.8	-61.8 (p=0.029)
	Medical (excl. Cardiology & Cancer)	4 weeks before change-point	6411.6	6124.5	-287.1 (p=0.029)
		4 weeks after change-point	6517.6	6132.5	-385.1 (p=0.029)
		4 weeks before end date	6696.5	6078.2	-618.3 (p=0.029)
	Paediatrics (medical)	4 weeks before change-point	1155.5	802	-353.5 (p=0.029)
		4 weeks after change-point	1191.6	734.5	-457.1 (p=0.029)
		4 weeks before end date	1270	744.8	-525.2 (p=0.029)
	Paediatrics (surgical)	4 weeks before change-point	39.2	58	18.8 (p=0.029)
		4 weeks after change-point	39.5	56.2	16.7 (p=0.057)
		4 weeks before end date	46.2	46	-0.2 (p=1)
	Surgery	4 weeks before change-point	3090.8	2766	-324.8 (p=0.029)
		4 weeks after change-point	3022.1	2603.5	-418.6 (p=0.029)
		4 weeks before end date	2983.6	2545	-438.6 (p=0.029)
*Difference column colours refer to whether the means were statistically higher (green), lower (red) or not different (yellow).					

Table S5: Planned hospital admissions - mean differences between 2018-2019 average and 2020-201 for 4 weekly time periods of 4 weeks before change-point (weeks ending 9 September to 27 September 2020), 4 weeks after change-point (weeks ending 4 October to 1 November 2020) and 4 weeks before end date (weeks ending 28 February to 28 March 2021). Stratified by the different characteristics.

Characteristic	Level	4 week period	2018-2019 Average Mean	2020-2021 Mean	Difference (p-value)*
Total		4 weeks before change-point	3562.2	2651.8	-910.4 (p=0.029)
		4 weeks after change-point	3367.1	2538.8	-828.3 (p=0.029)
		4 weeks before end date	3576.5	2508.2	-1068.3 (p=0.029)
Age	Aged under 5	4 weeks before change-point	278.8	245.5	-33.3 (p=0.057)
		4 weeks after change-point	267.9	212.8	-55.1 (p=0.029)
		4 weeks before end date	264.6	235	-29.6 (p=0.2)
	Aged 5 to 14	4 weeks before change-point	161.1	120.8	-40.3 (p=0.029)
		4 weeks after change-point	156.4	124.5	-31.9 (p=0.029)
		4 weeks before end date	155	134	-21 (p=0.114)
	Aged 15 to 44	4 weeks before change-point	614.8	443	-171.8 (p=0.029)
		4 weeks after change-point	559.6	420	-139.6 (p=0.029)
		4 weeks before end date	595.9	440.2	-155.7 (p=0.029)
	Aged 45 to 64	4 weeks before change-point	1018	736.8	-281.2 (p=0.029)
		4 weeks after change-point	959.2	717.5	-241.7 (p=0.029)
		4 weeks before end date	1033.8	703.5	-330.3 (p=0.029)
	Aged 65 to 84	4 weeks before change-point	633.8	469.9	-163.9 (p=0.005)
		4 weeks after change-point	610.4	451.1	-159.3 (p=0.005)
		4 weeks before end date	636.4	420.6	-215.8 (p=<0.001)
	Aged 85 and over	4 weeks before change-point	222.1	166	-56.1 (p=0.029)
		4 weeks after change-point	203.1	161.8	-41.3 (p=0.114)
		4 weeks before end date	254.5	154.2	-100.3 (p=0.029)
Sex	Female	4 weeks before change-point	1729.9	1263	-466.9 (p=0.029)
		4 weeks after change-point	1623	1265.8	-357.2 (p=0.029)
		4 weeks before end date	1773.2	1282.8	-490.4 (p=0.029)
	Male	4 weeks before change-point	1723.6	1272.2	-451.4 (p=0.029)
		4 weeks after change-point	1633.5	1183.2	-450.3 (p=0.029)
		4 weeks before end date	1734.5	1219.8	-514.7 (p=0.029)
SIMD	Quintile 1 - most deprived	4 weeks before change-point	818.1	625	-193.1 (p=0.029)
		4 weeks after change-point	789.2	597	-192.2 (p=0.029)
		4 weeks before end date	828.5	631.8	-196.7 (p=0.029)
	Quintile 2	4 weeks before change-point	758.5	579.8	-178.7 (p=0.029)
		4 weeks after change-point	707.6	531.8	-175.8 (p=0.029)
		4 weeks before end date	758.2	535.8	-222.4 (p=0.029)
	Quintile 3	4 weeks before change-point	735.8	538.5	-197.3 (p=0.029)
		4 weeks after change-point	699.2	529.2	-170 (p=0.029)
		4 weeks before end date	747.1	485.5	-261.6 (p=0.029)
	Quintile 4	4 weeks before change-point	666.4	463.5	-202.9 (p=0.029)
		4 weeks after change-point	600.5	464.2	-136.3 (p=0.029)
		4 weeks before end date	654.4	439.5	-214.9 (p=0.029)
	Quintile 5 - least deprived	4 weeks before change-point	568.9	438.5	-130.4 (p=0.029)
		4 weeks after change-point	558.8	413	-145.8 (p=0.029)
		4 weeks before end date	581.1	408.5	-172.6 (p=0.029)
Specialty	Cancer	4 weeks before change-point	118.9	109.8	-9.1 (p=0.468)
		4 weeks after change-point	125.1	104.8	-20.3 (p=0.028)
		4 weeks before end date	116	127.5	11.5 (p=0.343)
	Cardiology	4 weeks before change-point	123.2	114.2	-9 (p=0.11)
		4 weeks after change-point	127.5	96.8	-30.7 (p=0.029)
		4 weeks before end date	142.1	112.2	-29.9 (p=0.029)
	Community	4 weeks before change-point	54.9	48.5	-6.4 (p=0.343)
		4 weeks after change-point	56.2	40.5	-15.7 (p=0.029)
		4 weeks before end date	67.8	53.5	-14.3 (p=0.029)

Characteristic	Level	4 week period	2018-2019 Average Mean	2020-2021 Mean	Difference (p-value)*
	Gynaecology	4 weeks before change-point	235.9	150.2	-85.7 (p=0.029)
		4 weeks after change-point	219.8	137.8	-82 (p=0.029)
		4 weeks before end date	246.6	140.8	-105.8 (p=0.029)
	Medical (excl. Cardiology & Cancer)	4 weeks before change-point	831.6	631.8	-199.8 (p=0.029)
		4 weeks after change-point	733	569	-164 (p=0.029)
		4 weeks before end date	787.5	582.5	-205 (p=0.029)
	Paediatrics (medical)	4 weeks before change-point	235.8	216.2	-19.6 (p=0.2)
		4 weeks after change-point	230	186.2	-43.8 (p=0.029)
		4 weeks before end date	210.8	217.5	6.7 (p=0.686)
	Paediatrics (surgical)	4 weeks before change-point	36	23.2	-12.8 (p=0.029)
		4 weeks after change-point	33.8	23.8	-10 (p=0.058)
		4 weeks before end date	40.1	22.5	-17.6 (p=0.029)
	Surgery	4 weeks before change-point	1916.9	1346.5	-570.4 (p=0.029)
		4 weeks after change-point	1830.9	1372.2	-458.7 (p=0.029)
		4 weeks before end date	1953.6	1227.2	-726.4 (p=0.029)

*Difference column colours refer to whether the means were statistically higher (green), lower (red) or not different (yellow).

Table S6: Change in slope after reintroduced restrictions (intervention) on 22 September 2020 by age groups, with 95% confidence intervals

Outcome	Age group	Change in trend after intervention
A&E attendances	Under 5	-0.2 (-0.3, -0.1)
	5 to 14	-0.6 (-0.7, -0.5)
	15 to 44	-0.3 (-0.3, -0.2)
	45 to 64	-0.2 (-0.3, -0.2)
	65 to 84	-0.2 (-0.3, -0.2)
	85 and over	-0.2 (-0.3, -0.1)
Emergency hospital admissions	Under 5	-0.2 (-0.3, -0.1)
	5 to 14	-0.5 (-0.6, -0.4)
	15 to 44	-0.2 (-0.2, -0.1)
	45 to 64	-0.2 (-0.2, -0.1)
	65 to 84	-0.2 (-0.3, -0.2)
	85 and over	-0.2 (-0.2, -0.1)
Planned hospital admissions	Under 5	-0.2 (-0.3, -0.1)
	5 to 14	-0.3 (-0.4, -0.1)
	15 to 44	-0.3 (-0.4, -0.2)
	45 to 64	-0.3 (-0.4, -0.3)
	65 to 84	-0.4 (-0.4, -0.3)
	85 and over	-0.4 (-0.5, -0.3)
